# Association Between Systemic Inflammation and Radiographic Severity in Knee Osteoarthritis: A Trial Baseline Cohort Analysis

**DOI:** 10.7759/cureus.93371

**Published:** 2025-09-27

**Authors:** Abins TK, Ujwal Veluguleti, Nagma Sheenam, Sarankumar G, Neeru Gaur, Arindam Ghosh, Rejuwan Hussain, Nitesh Gonnade, Ravi Gaur

**Affiliations:** 1 Physical Medicine and Rehabilitation, All India Institute of Medical Sciences, Jodhpur, Jodhpur, IND; 2 Anaesthesiology, Fortis Escorts hospital, Jaipur, Jaipur, IND

**Keywords:** c-reactive protein (crp), crp-to-albumin ratio (car), erythrocyte sedimentation rate (esr), inflammatory biomarkers, kellgren-lawrence grade, knee osteoarthritis, monocyte-to-lymphocyte ratio (mlr), neutrophil-to-lymphocyte ratio (nlr), platelet-to-lymphocyte ratio (plr), radiographic severity

## Abstract

Background

Knee osteoarthritis (OA) is a common cause of pain and disability. Blood-based inflammatory markers may provide additional information on disease severity, but their clinical value is uncertain.

Objectives

To evaluate the association between inflammatory blood markers and radiographic severity of knee OA, and to assess their diagnostic performance.

Methods

In this cross-sectional study, 112 adults with radiographically confirmed knee OA [Kellgren-Lawrence (KL) grades I-III] were assessed. Demographic, hematological, and biochemical parameters were recorded. The Kruskal-Wallis test was used for between-group comparisons, Spearman’s rank for correlations, ordinal logistic regression for predictors, and receiver operating characteristic (ROC) curves for diagnostic accuracy.

Results

Significant differences across KL grades were observed for monocyte count, neutrophil count, neutrophil-to-lymphocyte ratio (NLR), monocyte-to-lymphocyte ratio (MLR), erythrocyte sedimentation rate (ESR), and vitamin D (all p < 0.05). However, correlation analysis revealed only a weakly significant association between KL grade and ESR (ρ = 0.241, p = 0.010), while other markers showed no significant correlation. Logistic regression identified ESR as the only independent predictor [odds ratio (OR) 1.046, 95% confidence interval (CI): 1.013-1.083, p = 0.008]. ROC analysis showed fair discrimination for ESR [area under the curve (AUC) = 0.646], C-reactive protein (CRP) (AUC = 0.662), and CRP-to-albumin ratio (CAR) (AUC = 0.685), whereas NLR, MLR, and platelet-to-lymphocyte ratio (PLR) were not significant.

Conclusions

ESR was the only inflammatory marker consistently associated with radiographic severity of knee OA. Other indices demonstrated limited diagnostic value, indicating that evaluation of OA severity should not rely solely on hematological ratios.

## Introduction

Knee osteoarthritis (OA) is a chronic, progressive musculoskeletal disorder and a major contributor to global disability, particularly in aging populations. It is histopathologically characterized by articular cartilage degradation, synovial inflammation, osteophyte formation, and subchondral bone remodeling [1]. Although the Kellgren-Lawrence (KL) radiographic grading system remains the gold standard for assessing OA severity, it primarily reflects structural changes and does not capture the complex biochemical and inflammatory processes that underlie symptom burden and disease progression [2].

Emerging evidence implicates chronic, low-grade inflammation-both local and systemic-as a central mechanism in osteoarthritis (OA) pathogenesis, with innate immune dysregulation contributing to joint tissue degeneration and pain sensitization. Proinflammatory cytokines and systemic inflammatory markers, including acute-phase proteins and potentially hematological ratios, are being explored as surrogate indicators of disease activity and progression [3]. Ratios such as the monocyte-to-lymphocyte ratio (MLR), neutrophil-to-lymphocyte ratio (NLR), and platelet-to-lymphocyte ratio (PLR) are inexpensive, routinely available blood parameters that reflect systemic inflammation and immune response status [4]. Elevated MLR and NLR have been observed in patients with more advanced OA, although their standalone diagnostic utility is considered modest [5].

Beyond cellular ratios, biochemical markers involved in the OA inflammatory cascade are linked to persistent pain phenotypes and may aid in symptomatic stratification [6]. Likewise, general inflammatory markers such as C-reactive protein (CRP) and erythrocyte sedimentation rate (ESR) have been variably linked to OA severity [7]. However, inconsistencies in their prognostic performance and the influence of comorbidities limit their clinical reliability. While synovial fluid markers offer more joint-specific insight, their invasiveness hinders their utility in everyday clinical settings.

Despite increasing interest in inflammatory markers in knee osteoarthritis (OA), their clinical utility remains limited due to methodological heterogeneity and poor integration with structural imaging. Notably, MRI features like synovitis and effusion correlate more strongly with neuropathic pain than radiographic KL grades, highlighting the need for comprehensive assessment approaches. This cross-sectional study examines the relationship between hematological markers (MLR, NLR, PLR), CRP-to-albumin ratio (CAR), vitamin D, CRP, and ESR with radiographic OA severity. Using correlation analysis, ordinal logistic regression, and receiver operating characteristic (ROC) curves, we evaluate their diagnostic value and potential as adjunct tools for more personalized disease stratification.

## Materials and methods

This cross-sectional observational analysis was conducted at a tertiary care academic center between December 2024 and March 2025. The data were derived from the baseline (first visit) assessment of participants enrolled in an ongoing randomized controlled trial (RCT) cohort evaluating interventions in knee osteoarthritis (CTRI/2025/05/087473). Ethical approval for the study was obtained from the Institutional Ethics Committee, AIIMS Jodhpur. Written informed consent was obtained from all participants before enrollment. The study adhered to the ethical principles outlined in the Declaration of Helsinki and followed the Strengthening the Reporting of Observational Studies in Epidemiology (STROBE) guidelines.

The sample size of 112 participants was considered adequate for ordinal logistic regression, following the guideline of 10-20 participants per predictor variable. With around 10 predictors (e.g., MLR, NLR, CRP, ESR), the sample size aligns with recommendations, ensuring stable parameter estimation. Although larger samples may be needed for smaller effect sizes, our design is consistent with similar cross-sectional OA studies

Adults aged 18 years or older with a radiographically confirmed diagnosis of knee osteoarthritis (KL Grades I-III), experiencing moderate to severe knee pain, and able to walk unassisted or with minimal assistance were included. Patients with KL Grade IV osteoarthritis, inflammatory arthropathies such as rheumatoid arthritis, active infection, recent major trauma or surgery, known malignancy, or those on immunosuppressive therapy were excluded. Patients with chronic liver disease, renal failure, or other systemic autoimmune disorders were also excluded.

All participants underwent standard anteroposterior and lateral knee radiographs, and OA severity was graded using the KL classification. Demographic variables, including age, sex, and body mass index (BMI), were recorded. Hematological parameters (monocyte, neutrophil, lymphocyte, and platelet counts), inflammatory markers [C-reactive protein (CRP), erythrocyte sedimentation rate (ESR)], and biochemical markers (serum albumin, vitamin D levels) were measured. Derived inflammatory ratios were calculated as follows: MLR = monocyte/lymphocyte, NLR = neutrophil/lymphocyte, PLR = platelet/lymphocyte, and CAR = CRP/albumin.

Descriptive statistics were used to summarize participant characteristics across KL grades. The Kruskal-Wallis test was applied to compare inflammatory and biochemical markers between KL grades. Spearman’s rank correlation coefficient was used to assess associations between KL grade and continuous variables. Ordinal logistic regression was performed to identify predictors of higher KL grade severity. Model fit was assessed using McFadden’s and Nagelkerke’s R². Finally, receiver operating characteristic (ROC) curve analysis was conducted to evaluate the discriminatory ability of each marker, with area under the curve (AUC) and 95% confidence intervals reported. Statistical significance was set at p < 0.05. All analyses were conducted using SPSS version 23 (IBM Corp., Armonk, New York, USA).

## Results

In the present study, the mean age of female participants (n = 59) was 57.3 years (SD = 10.16), while the mean age of male participants (n = 53) was 56.6 years (SD = 8.22). Table 1 summarizes the demographic, hematological, biochemical, and derived inflammatory indices across the three Kellgren-Lawrence (KL) grades of knee osteoarthritis. Between-group comparisons revealed statistically significant differences in monocyte count (p < 0.001), neutrophil count (p < 0.001), NLR (p < 0.001), MLR (p < 0.001), ESR (p = 0.023), and vitamin D (p = 0.012) across KL grade groups. However, despite these significant between-group differences, most of these markers did not show a significant monotonic correlation with the ordered KL grades, suggesting a non-linear relationship or that the differences are primarily driven by the separation between KL Grade I and the higher grades. No significant differences were observed for age, BMI, lymphocyte count, platelet count, CRP, albumin, PLR, or CAR.

**Table 1:** Comparison of Hematological and Biochemical Parameters Across KL Grades in Patients with Osteoarthritis KL: Kellgren-Lawrence; IQR: interquartile range; BMI: body mass index; NLR: neutrophil-to-lymphocyte ratio; MLR: monocyte-to-lymphocyte ratio; CRP: C-reactive protein; ESR: erythrocyte sedimentation rate; PLR: platelet-to-lymphocyte ratio; CAR: CRP-to-albumin ratio.

Parameter	KL Grade	Median	IQR	25th Percentile	75th Percentile	p value	Chi-square
Age	II	59	14.25	49.75,	64	0.783	0.488
	III	55	16	49.5,	65.5		
	I	55	5	53,	58		
BMI	II	27.7	3.35	25.675,	29.025	0.625	0.939
	III	27.4	3.25	25.6,	28.85		
	I	25.7	3.6	23.7,	27.3		
Monocyte	II	0.53	0.2275	0.407,	0.635	<0.001	26.715
	III	0.64	0.235	0.525,	0.76		
	I	0.23	0.05	0.23,	0.28		
Neutrophil	II	4.025	2.2925	2.94,	5.232	<0.001	23.459
	III	5.14	1.505	4.265,	5.77		
	I	2.12	0.13	2.1,	2.23		
Lymphocyte	II	2.615	1.35	2.008,	3.357	0.787	0.479
	III	2.67	1.225	1.94,	3.165		
	I	2.19	0.07	2.13,	2.2		
Platelet	II	282.5	101	222.25,	323.25	0.363	2.024
	III	253	139.5	202,	341.5		
	I	302	68	269,	337		
NLR	II	1.634	1.2839	1.193	2.477	<0.001	15.043
	III	1.979	1.0833	1.536	2.619		
	I	0.964	0.4796	0.63	1.11		
MLR	II	0.184	0.12	0.153	0.273	<0.001	17.529
	III	0.222	0.1338	0.201	0.334		
	I	0.127	0.0234	0.108	0.131		
CRP	II	4.795	6.0975	2.328	8.425	0.863	0.296
	III	5.53	3.655	3.465	7.12		
	I	5.03	5.76	3	8.76		
Albumin	II	4.04	1.01	3.5	4.51	0.923	0.16
	III	4.13	0.835	3.65	4.485		
	I	4.21	0.59	3.71	4.3		
ESR	II	23	29.25	10.75	40	0.023	7.56
	III	32	14	27	41		
	I	31	16	22	38		
Vitamin D	II	26.6	11.525	19.2	30.725	0.012	8.898
	III	21.5	19.45	10.55	30		
	I	32.8	9.8	28.4	38.2		
PLR	II	111.5	68.0475	81.553	149.6	0.266	2.647
	III	100	60.025	72.075	132.1		
	I	122.83	52.12	120.45	172.57		
CAR	II	1.105	1.34	0.565	1.905	0.44	1.64
	III	1.27	0.865	0.86	1.725		
	I	2	0.84	1.19	2.03		

**Figure 1:**
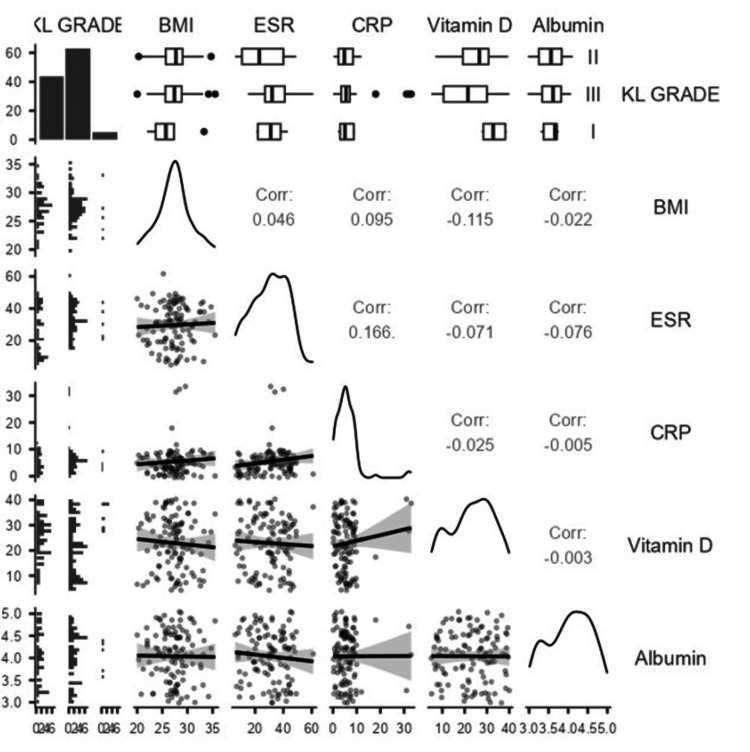
Pairwise Correlations Between KL Grade and Clinical Biomarkers in OA Patients KL: Kellgren-Lawrence; BMI: body mass index; ESR: erythrocyte sedimentation rate; CRP: C-reactive protein.

Spearman’s rank correlation analysis (Figure 1) demonstrated a weak but statistically significant positive association between KL grade and ESR (ρ = 0.241, p = 0.010), indicating a mild link between radiographic severity and systemic inflammation. In contrast, KL grade showed no significant monotonic correlation with BMI, CRP, vitamin D, albumin, or hematological counts (monocyte ρ = 0.182, p = 0.055; neutrophil ρ = 0.139, p = 0.145; lymphocyte ρ = -0.008, p = 0.931; platelet ρ = 0.014, p = 0.880). Thus, although some markers (monocyte, neutrophil, NLR, MLR) differed significantly between groups in Table 1, their correlations with KL grade as an ordinal variable were weak or non-significant.

**Figure 2:**
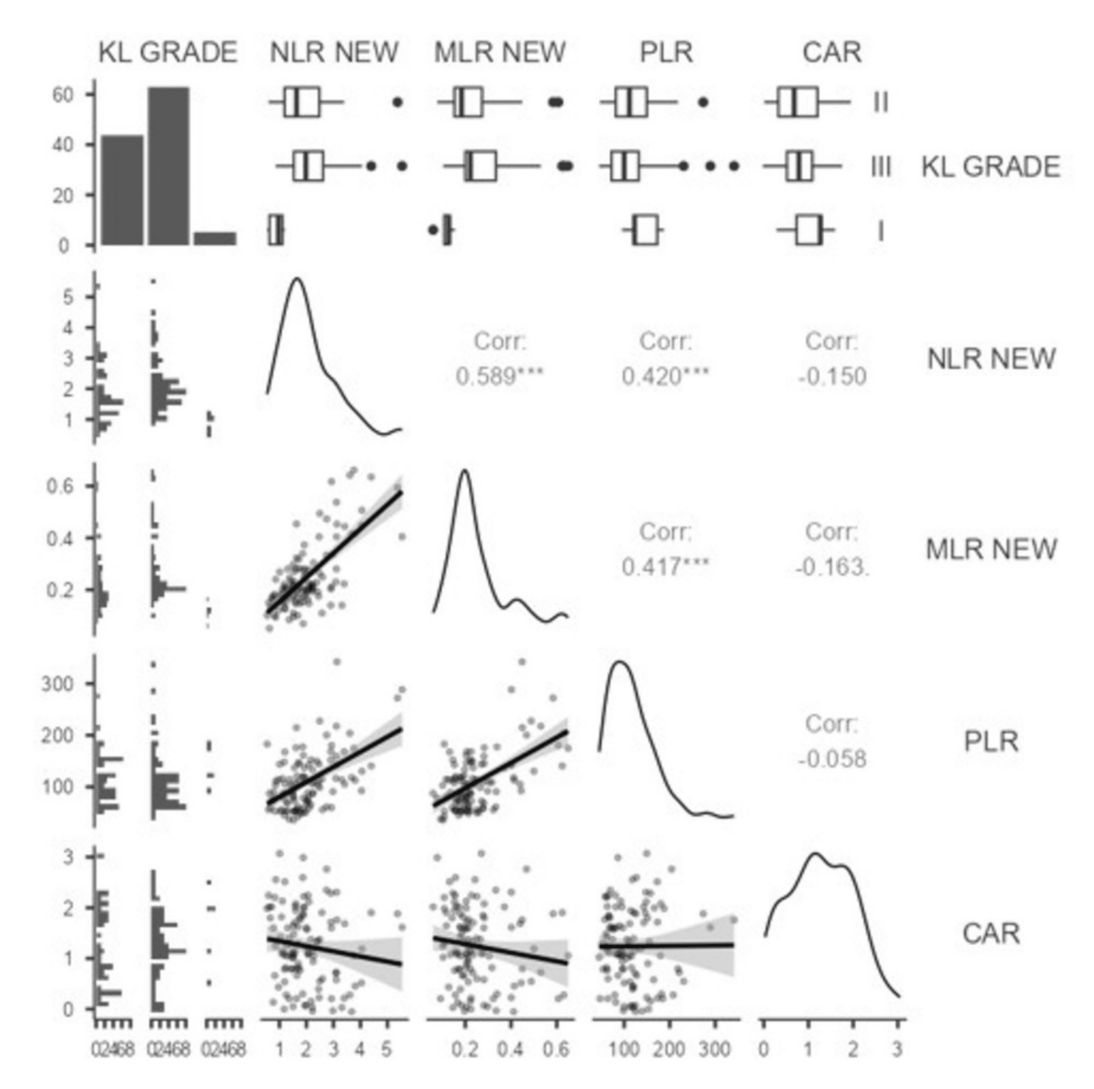
Correlation Matrix and Distributions of KL Grade With Inflammatory Ratios in OA Patients KL: Kellgren-Lawrence; NLR: neutrophil-to-lymphocyte ratio; MLR: monocyte-to-lymphocyte ratio; PLR: platelet-to-lymphocyte ratio; CAR: CRP-to-albumin ratio.

Among inter-parameter relationships, monocytes showed weak positive correlations with neutrophils (ρ = 0.159, p = 0.094) and lymphocytes (ρ = 0.051, p = 0.590). Neutrophil count was weakly but significantly negatively correlated with platelet count (ρ = -0.191, p = 0.044). For inflammatory indices (Figure 2), MLR correlated strongly with NLR (ρ = 0.589, p < 0.001) and PLR (ρ = 0.417, p < 0.001), and PLR correlated with NLR (ρ = 0.420, p < 0.001). CAR did not show significant correlations with other markers.

An ordinal logistic regression model (Table 2) was fitted to predict radiographic osteoarthritis severity using inflammatory biomarkers. The model showed modest explanatory power (Nagelkerke R² = 0.070; deviance = 176.0; AIC = 192.0). Only ESR emerged as a statistically significant predictor [OR = 1.046, 95% CI (1.013-1.083), p = 0.008]. Each unit increase in ESR was associated with a 4.6% higher likelihood of progressing to a more severe KL grade. All other biomarkers were non-significant predictors, with wide confidence intervals (e.g., MLR 95% CI -3.64 to 5.63), highlighting estimation uncertainty.

**Table 2:** Logistic Regression Analysis of Inflammatory Biomarkers Associated with Kellgren-Lawrence (KL) Grade in Osteoarthritis Patients CI: confidence interval; SE: standard error; NLR: neutrophil-to-lymphocyte ratio; MLR: monocyte-to-lymphocyte ratio; CRP: C-reactive protein; ESR: erythrocyte sedimentation rate; PLR: platelet-to-lymphocyte ratio; CAR: CRP-to-albumin ratio.

Predictor	Estimate	Lower 95% CI	Upper 95% CI	SE	Z	p-value	Odds Ratio
NLR	0.01809	-0.5472	0.59004	0.28834	0.06273	0.95	1.018
MLR	0.98932	-3.637	5.62512	2.34929	0.42112	0.674	2.689
CRP	0.02764	-0.0513	0.11099	0.04169	0.66302	0.507	1.028
ESR	0.04516	0.0127	0.0794	0.01694	2.66627	0.008	1.046
PLR	-0.00078	-0.0102	0.00857	0.00476	-0.16366	0.87	0.999
CAR	0.00133	-0.6095	0.61027	0.31031	0.00427	0.997	1.001

**Figure 3:**
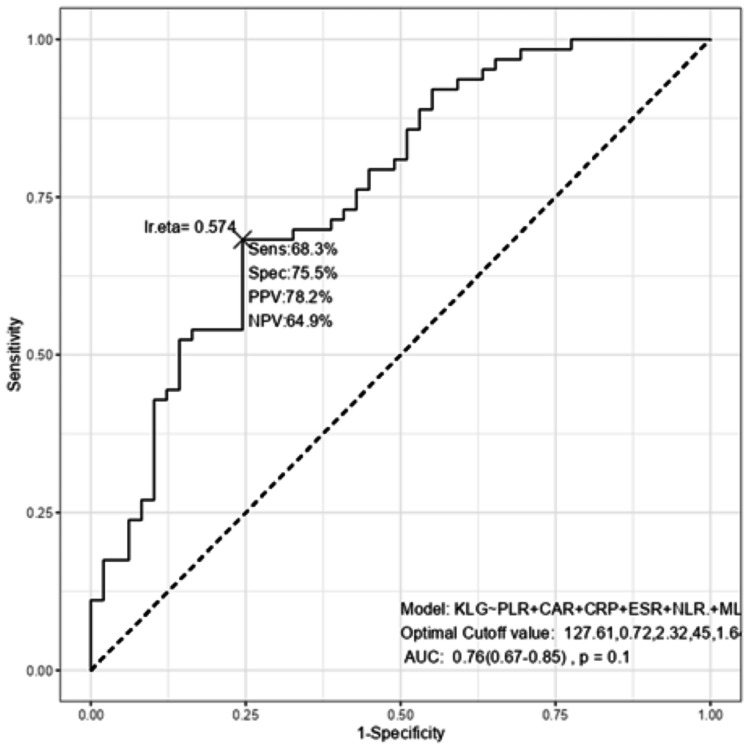
Receiver Operating Characteristic (ROC) Curve for Inflammatory Biomarkers Predicting Kellgren-Lawrence (KL) Grade Severity in Osteoarthritis PPV: positive predictive value; NPV: negative predictive value; KLG: Kellgren-Lawrence grade; PLR: platelet-to-lymphocyte ratio; CAR: CRP-to-albumin ratio; CRP: C-reactive protein; ESR: erythrocyte sedimentation rate; NLR: neutrophil-to-lymphocyte ratio; MLR: monocyte-to-lymphocyte ratio; AUC: area under the curve.

**Figure 4:**
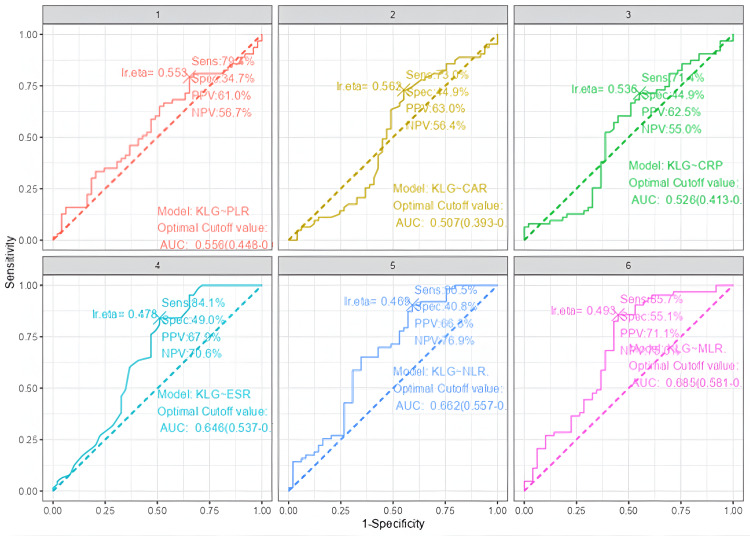
Diagnostic Performance of Individual Inflammatory Biomarkers in Predicting Kellgren-Lawrence (KL) Grade Severity in Osteoarthritis Patients PPV: positive predictive value; NPV: negative predictive value; KLG: Kellgren-Lawrence grade; PLR: platelet-to-lymphocyte ratio; AUC: area under the curve; CAR: CRP-to-albumin ratio; CRP: C-reactive protein; ESR: erythrocyte sedimentation rate; NLR: neutrophil-to-lymphocyte ratio; MLR: monocyte-to-lymphocyte ratio.

Receiver operating characteristic (ROC) curves (Figures 3, 4) were generated for six inflammatory markers. ESR (AUC = 0.646, p = 0.004), CRP (AUC = 0.662, p = 0.001), and CAR (AUC = 0.685, p < 0.001) demonstrated statistically significant discriminatory ability for KL severity, whereas PLR (AUC = 0.556, p = 0.156), NLR (AUC = 0.507, p = 0.452), and MLR (AUC = 0.526, p = 0.328) did not. Pairwise AUC comparisons showed CAR significantly outperformed MLR (AUC difference = -0.1785, 95% CI -0.3470 to -0.0099, p = 0.038), and ESR outperformed MLR (AUC difference = -0.1390, 95% CI -0.2707 to -0.0072, p = 0.039). However, the overall DeLong test across all six markers was not significant (Z = 4.38, p = 0.496).

## Discussion

Our cross-sectional analysis of 112 knee OA patients revealed that among various inflammatory markers, only ESR showed a significant association with radiographic severity (KL grades I-III). In contrast, classical systemic inflammatory indices like CRP and the CRP-to-albumin ratio (CAR) demonstrated only fair discriminative ability for higher KL grades (AUCs in the “fair” range) and did not significantly predict radiographic severity. Similarly, composite blood cell-derived indices (NLR, MLR, PLR), despite moderate intercorrelations indicative of a common inflammatory milieu, failed to meaningfully differentiate radiographic OA severity. These findings align with emerging evidence that while markers such as NLR and MLR may reflect an underlying inflammatory response in OA, they lack diagnostic utility for assessing structural severity or prognosis. Our findings align with recent evidence showing that elevated MLR and NLR modestly correlate with advanced KL grades but lack reliability as standalone markers of disease severity [6]. Taken together, our findings highlight the limitations of KL grading in capturing the inflammatory component of OA, reinforcing the need for a more comprehensive assessment beyond radiographic staging.

The disconnect between structural severity and systemic inflammation in OA has important implications. Radiographic KL grading is a time-honored tool for staging knee OA, but it primarily captures late-stage structural damage (osteophytes, joint space narrowing) and overlooks the biological heterogeneity of the disease. Prior research has shown, for example, that serum CRP - a prototypical inflammation marker - correlates with OA pain and functional impairment but not with radiographic grades [7]. This suggests that radiographic severity often fails to reflect the low-grade systemic inflammation present in many OA patients. Clinically, it is well recognized that symptom severity can diverge from X-ray findings: some patients with mild KL changes experience intense pain and effusions, whereas others with advanced changes report surprisingly little pain. Indeed, pain intensity in knee OA is largely unrelated to KL grade in many cases [8]. Our finding that most blood inflammatory markers were not associated with KL grade further highlights this structure-inflammation discordance. In essence, Kellgren-Lawrence grade alone is an inadequate proxy for an “inflammatory phenotype” of OA, as it does not account for synovial activation, systemic immune-response, or pain mechanisms. This underscores a key limitation of traditional radiographic assessment: it represents a common end-stage outcome of various pathological pathways, without distinguishing the route by which a patient arrived at that outcome [9].

In light of this inadequacy, there is a growing consensus that we must move beyond one-dimensional assessments and embrace a multidimensional framework for OA phenotyping. Osteoarthritis is no longer viewed as a homogeneous “wear-and-tear” degeneration of cartilage; rather, it is appreciated as a complex disease with inflammatory, metabolic, and mechanical components interacting in a patient-specific manner [6]. Recent research trends strongly support the existence of distinct OA subsets (or endotypes), including an “inflammatory phenotype” characterized by elevated systemic cytokines, synovial inflammation, and possibly metabolic dysregulation. For instance, unsupervised clustering of OA patients by metabolic and inflammatory profiles has identified multiple phenotypes of knee OA with clear clinical relevance. OA phenotypes based on inflammation and metabolic status show distinct pain and progression patterns, highlighting the need for a composite approach beyond radiographs to capture disease complexity [10].

We propose a multidimensional framework for OA assessment that integrates biomarkers, advanced imaging, and clinical indices, moving beyond KL-grade limitations. Given OA's complex, multi-tissue pathology, no single biomarker suffices. Instead, a composite inflammatory index-similar to RA’s multi-biomarker scores-may better detect and predict disease progression by capturing subtle systemic inflammation [9].

Second, advanced imaging modalities should be leveraged to characterize inflammation and tissue pathology that plain radiographs miss. MRI and musculoskeletal ultrasound have emerged as valuable tools to visualize synovitis, effusions, cartilage integrity, and bone marrow lesions in OA [11]. Inflammatory changes on imaging (such as synovial thickening or power Doppler signal on ultrasound) might identify an “inflammatory OA” subset even when CRP/ESR levels are normal. Notably, ultrasound offers dynamic, real-time assessment of both structural and inflammatory changes, making it a promising adjunct to clinical examination. By determining a patient’s predominant “ultrasound phenotype” - for example, synovitis-dominant vs. cartilage-loss-dominant - clinicians could better tailor therapies. This concept of an “ultrasound phenotype-based approach” recognizes that different tissue pathologies (synovial membrane vs. bone vs. cartilage) may drive disease in different patients, and aligning treatment with the dominant pathology could improve outcomes. In short, incorporating modern imaging can enhance our phenotypic classification beyond the bony changes captured by KL grade [12].

A multidimensional OA framework should integrate clinical symptoms, biomarkers, and imaging to better reflect disease activity. Standardized tools like Western Ontario and McMaster Universities Osteoarthritis Index (WOMAC) or Knee Injury and Osteoarthritis Outcome Score (KOOS) can capture symptom variations linked to biological differences (e.g., inflammatory vs. mechanical pain). This unified assessment could yield a disease severity score, improving both patient stratification and treatment targeting. Translationally, such an approach may enhance trial design by selecting phenotypically homogeneous patients, potentially reversing Disease-Modifying Osteoarthritis Drug (DMOAD) trial failures. Clinically, it enables personalized care-anti-inflammatory therapies for inflammatory phenotypes, biomechanical interventions for others-and supports closer monitoring of high-risk individuals. As in cardiology or rheumatology, OA biomarkers may soon enter routine use, offering public health gains through improved prognostication and targeted management [7,9,12].

This study acknowledges several important limitations. The cross-sectional design restricts the ability to infer causality and prevents assessment of temporal changes in biomarker levels. The moderate sample size limits statistical power, particularly for multivariable regression models. Details of KL grading procedure, assessor blinding, and inter-rater reliability were not specified, and the assay methodology for CRP, albumin, and vitamin D was not fully described, which may affect reproducibility across settings. Many systemic biomarkers analyzed are nonspecific and may be confounded by comorbidities such as obesity or metabolic syndrome, and KL grading itself has limited sensitivity and reliability as a surrogate for local joint inflammation. The absence of strong biomarker-KL correlations likely reflects both the inadequacy of radiographic severity as a proxy for inflammatory burden and potential non-linear relationships that were not fully explored. Finally, generalizability is limited by the single-center design and lack of inclusion of end-stage (KL IV) OA cases.

Still, the findings support growing evidence that OA is not merely a mechanical or radiographic disorder but a complex, systemic disease with inflammatory and metabolic components. We advocate for a shift toward a multidimensional assessment framework integrating advanced imaging, systemic/local biomarkers, and standardized clinical symptom profiling (e.g., WOMAC, KOOS). This approach could yield composite disease activity scores, enable precise phenotyping, and facilitate tailored interventions.

Such a framework would enhance research by improving patient selection in clinical trials-potentially rescuing failed DMOAD efforts-and could transform clinical care through personalized treatment strategies based on inflammatory or mechanical phenotypes. Future priorities include validating composite biomarker algorithms, developing predictive models for progression or surgery, testing phenotype-based therapies, and demonstrating cost-effectiveness. As international groups call for refined phenotyping and biospecimen integration, this paradigm shift promises to move OA management beyond KL-centric assessment toward a personalized, biomarker-informed future. While this study focused on systemic inflammation in a common degenerative joint disease, the same biomarker-based methodologies could be applied to investigate other conditions characterized by aberrant inflammatory or bone-forming responses. Markers such as ESR and NLR may provide insights into the local microenvironment that predisposes to atypical pathological tissue changes [13].

## Conclusions

In this cross-sectional analysis of knee OA patients, ESR emerged as the only inflammatory marker independently associated with radiographic severity. CRP and CAR demonstrated fair but likely insufficient discriminatory value for clinical application (AUC ~0.66-0.69, p > 0.05 in regression), while hematological ratios such as NLR, MLR, and PLR did not demonstrate clinical utility. These findings underscore the discordance between systemic inflammation and KL grading, indicating that radiographs alone inadequately capture the biological complexity of OA.

A multidimensional assessment framework that integrates systemic biomarkers with advanced imaging and clinical symptom indices is needed to more accurately characterize disease phenotypes. Such an approach may improve patient stratification, inform individualized treatment strategies, and strengthen the design of future clinical trials targeting inflammatory subsets of OA.
